# A Dipping Duration Study for Optimization of Anodized-Aluminum Pressure-Sensitive Paint

**DOI:** 10.3390/s101109799

**Published:** 2010-11-02

**Authors:** Hirotaka Sakaue, Keiko Ishii

**Affiliations:** Aerospace Research and Development Directorate, Japan Aerospace Exploration Agency/Chofu, Tokyo 182-8522, Japan; E-Mail: kekoi@chofu.jaxa.jp

**Keywords:** anodized aluminum, pressure-sensitive paint, dipping duration

## Abstract

Anodized-aluminum pressure-sensitive paint (AA-PSP) uses the dipping deposition method to apply a luminophore on a porous anodized-aluminum surface. We study the dipping duration, one of the parameters of the dipping deposition related to the characterization of AA-PSP. The dipping duration was varied from 1 to 100,000 s. The properties characterized are the pressure sensitivity, temperature dependency, and signal level. The maximum pressure sensitivity of 65% is obtained at the dipping duration of 100 s, the minimum temperature dependency is obtained at the duration of 1 s, and the maximum signal level is obtained at the duration of 1,000 s, respectively. Among the characteristics, the dipping duration most influences the signal level. The change in the signal level is a factor of 8.4. By introducing a weight coefficient, an optimum dipping duration can be determined. Among all the dipping parameters, such as the dipping duration, dipping solvent, and luminophore concentration, the pressure sensitivity and signal level are most influenced by the dipping solvent.

## 1. Introduction

Anodized-aluminum pressure-sensitive paint (AA-PSP) is an optical sensor, which gives global information in unsteady flow measurements [1]. AA-PSP consists of a molecular pressure probe of a luminophore and an anodized aluminum as a supporting matrix. The luminophore is applied on the anodized-aluminum surface by the dipping deposition method [2]. This method requires a luminophore, a solvent, and an anodized-aluminum coating. The anodized coating is dipped in the luminophore solution to apply the luminophore on the coating surface. In previous reports the AA-PSP was optimized by controlling the dipping deposition parameters [2,3]. Sakaue reported the effects of solvent on the characteristics of AA-PSP [2]. By varying the solvent, he found that dichloromethane gave the best pressure sensitivity and signal level. Sakaue and Ishii controlled the luminophore concentration in the dipping deposition to optimize the AA-PSP characteristics [3]. They varied the luminophore concentration from 0.001 to 10 mM. A luminophore concentration of 0.1 mM provided optimum conditions for the pressure sensitivity, temperature dependency, and signal level. In these reports, a dipping duration in the dipping deposition was fixed at one hour.

The dipping duration can be another important parameter that influences the AA-PSP characteristics, because it would influence the amount of luminophore applied on the anodized-aluminum surface. The effects on the dipping duration as well as the above mentioned dipping parameters would give us fundamental knowledge to apply various luminophores on the anodized aluminum coating. In this paper, we varied the dipping duration related to the AA-PSP characterizations for optimizing AA-PSP. Steady-state characterizations are focused on the present study, because an unsteady-state characterization of response time was minimal related to the dipping deposition method [2]. These characterizations are the pressure sensitivity, temperature dependency, and signal level.

## 2. Experiment

We chose bathophen ruthenium (GFS Chemicals) as a conventional luminophore for AA-PSP. Based on the previous reports [2,3], dichloromethane was chosen as a solvent, and the luminophore solution concentration was fixed at 0.1 mM. The anodized coating thickness was 10 ± 1 μm measured by an eddy current apparatus (Kett, LZ-330). To study the effect of dipping duration, it was varied from 1 to 100,000 s. Table 1 lists the conditions of the AA-PSP preparation. Prepared AA-PSPs are labeled based on their dipping conditions, which are also listed in Table 1 as Sample ID. AAPSP_3600_ provides the same conditions reported in [3]. For each dipping condition, three samples were prepared to study the repeatability of dipping procedures.

We used a spectrometer combined with a pressure- and temperature-controlled chamber for AA-PSP characterizations. This spectrometer system characterizes the luminescent spectrum of an AA-PSP with varying pressures and temperatures. The excitation wavelength was set at 460 nm. The luminescent signal, *I*, was determined by the integration of AA-PSP spectrum from 600 to 700 nm. The test gas was dry air. Throughout all calibrations, the reference conditions were chosen at 100 kPa and 25 °C. All calibrations were averaged by three data sets prepared by the same dipping procedures. Standard deviation was calculated as an error bar. Details of the system can be found in [2]. The definition and procedures used to derive the characterizations are described in Sections 2.1. through 2.3.

### 2.1. Pressure Characterization

The pressure was controlled from 5 to 120 kPa with a constant temperature at 25 °C for the pressure characterization. Based on the Stern-Volmer relationship, the luminescent intensity, *I*, is related to a quencher [4]:

(1) $$\frac{I_{0}}{I}=1+K_{q}\left[O_{2}\right]$$

where the subscript *0* denotes the condition without the quencher and *K_q_* is the Stern-Volmer quenching constant. The quencher is oxygen, which is described by the oxygen concentration, [*O_2_*]. For AA-PSP, [*O_2_*] can be described by the adsorption and surface diffusion of the adsorbed oxygen on an anodized-aluminum surface. We can describe [*O_2_*] by the partial pressures of oxygen as well as the static pressures. These are combined with Equation (1) to give the adsorption-controlled model [5]:

(2) $$\frac{I_{\mathrm{ref}}}{I}=A+B{\left(\frac{p}{p_{\mathrm{ref}}}\right)}^{\gamma }$$

*A*, *B*, and *γ* are calibration constants. Here, *ref* denotes the reference conditions.

Pressure sensitivity, *σ*(%), describes the change in *I* over a given pressure change. This corresponds to a slope of Equation (2) at the reference conditions:

(3) $$\sigma ={\frac{d\left(I_{\mathrm{ref}}/I\right)}{d\left(p/p_{\mathrm{ref}}\right)}|}_{p=p_{\mathrm{ref}}}=B\cdot \gamma \;(\%)$$

To discuss the effects of *σ* on the dipping duration, it is normalized as follows:

(4) $$\mathrm{norm}\sigma =\frac{\sigma -\sigma_{\text{min}}}{\sigma_{\text{max}}-\sigma_{\text{min}}}$$

where *σ*_max_ and *σ*_min_ are the maximum and the minimum pressure sensitivities, respectively.

### 2.2. Temperature Characterization

For the temperature characterization, the temperature was controlled from 10 to 50 °C with a constant pressure at 100 kPa. This can be described as the third order polynomial in Equation (5):

(5) $$\frac{I}{I_{\mathrm{ref}}}=C_{0}+C_{1}T+C_{2}T^{2}+C_{3}T^{3}$$

Here, *C_0_*, *C_1_*, *C_2_*, and *C_3_* are calibration constants. We defined the temperature dependency, *δ*, which is a slope of the temperature calibration at the reference conditions. If the absolute value of *δ* is large, it tells us that the change in *I* over a given temperature change is also large. This is unfavorable condition as a pressure sensor. On the contrary, zero *δ* means AA-PSP is temperature independent, which is a favorable condition as a pressure sensor:

(6) $$\delta ={\frac{d\left(I/I_{\mathrm{ref}}\right)}{dT}|}_{T=T_{\mathrm{ref}}}=C_{1}+2C_{2}T_{\mathrm{ref}}+3C_{3}T_{\mathrm{ref}}{}^{2}\;(\%/{}^{\circ}C)$$

To discuss the effects of *δ* on the dipping duration, it is normalized as follows:

(7) $$\mathrm{norm}\delta =\frac{\delta -\delta_{\text{min}}}{\delta_{\text{max}}-\delta_{\text{min}}}$$

where *δ*_max_ and *δ*_min_ are the maximum and the minimum temperature dependencies.

### 2.3. Luminescent Signal Characterization

For the luminescent signal characterization, all the AA-PSPs were measured with the same optical setup in the spectrometer system but replacing the AA-PSP in the chamber at the reference conditions. We non-dimensionalized *I* by that of AAPSP_3600_, which is our reference AA-PSP. We call this as the signal level, *η*, shown in Equation (8):

(8) $$\eta =\frac{I}{I_{\mathrm{AAPSP}3600}}\;(\%)$$

where *I_AAPSP3600_* denotes *I* of AAPSP_3600_ at the reference conditions.

To discuss the effects of *η* on the dipping duration, it is normalized as follows:

(9) $$\mathrm{norm}\eta =\frac{\eta -\eta_{\text{min}}}{\eta_{\text{max}}-\eta_{\text{min}}}$$

Here, *η*_max_ and *η*_min_ are the maximum and the minimum signal levels.

## 3. Characterization Results

### 3.1. Pressure Sensitivity

**Figure 1.**
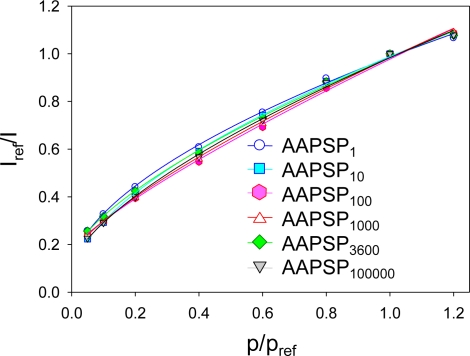
Pressure calibrations of AA-PSPs with varying the dipping duration.

Figure 1 shows the pressure calibrations of AA-PSPs. Calibration points were fitted with Equation (2). The value of *σ* was determined from Equation (3). The maximum *σ* of 65% and the minimum *σ* of 52% were obtained from AAPSP_100_ and AAPSP_1_, respectively. We prepared three samples for each dipping duration. The mean values are shown with their standard deviations as error bars (Table 2). When we consider the error, the difference in *σ* was around 60% for the dipping duration over 100 s. Even though the fifth order difference in the dipping duration was provided, a minimal effect was seen on the pressure sensitivity.

### 3.2. Temperature Dependency

**Figure 2.**
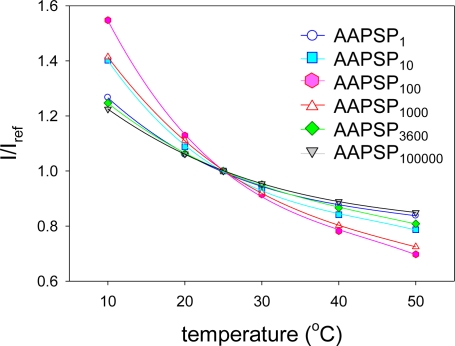
Temperature calibrations of AA-PSPs with varying the dipping duration.

Figure 2 shows the temperature calibrations of AA-PSPs. The calibrations were fitted with Equation (5). The temperature calibrations showed the decrease in *I* with increase temperature.

The value of *δ* was determined from Equation (6) (Table 2). With increase the dipping duration, we can see that *δ* decreased until 100 s and increased over this dipping duration. The difference of *δ* was roughly a factor of 2. The variation of *δ* was greater than that of the error bar. Compared to the effect on the pressure sensitivity, the dipping duration showed a greater effect on the temperature dependency.

### 3.3. Signal Level

The value of *η* was determined from Equation (8) (Table 2). There was a peak dipping duration to maximize *η*. The maximum *η* was obtained from AAPSP_1000_, whose dipping duration was 1,000 s. For a short dipping duration, the luminophore would remain in the luminophore solution instead of applying onto the anodized surface. Roughly, the difference of *η* was a factor of 8.5 by varying the dipping duration. Even though we increased the dipping duration over 1,000 s, *η* decreased. This may be due to the concentration quenching [4]. The variation of *η* was greater than that of the error bar. Compared to the effect on the pressure sensitivity and temperature dependency, the dipping duration showed the greatest effect on the signal level.

## 4. Discussion

### 4.1. Optimum Dipping Duration

**Figure 3.**
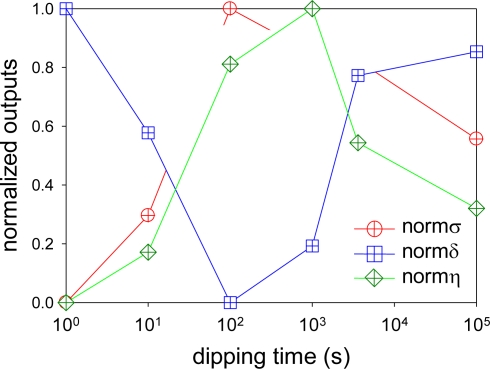
Normalized outputs of AA-PSP. The pressure sensitivity is shown as *normσ*, the temperature dependency as *normδ*, and the signal level as *normη*, respectively.

As a pressure sensor, we need *δ* to be close to zero or zero itself. At the same time, we need a higher *σ* as well as a higher *η* to give a higher luminescent output for a given pressure. These conditions match when all the normalized outputs in Figure 3 are the maximum. Unfortunately, the outputs did not show the desired case. The values of *normσ* and *normη* have similar trend but *normδ* is basically the opposite.

To determine an optimum dipping duration, we introduce weight coefficients, *α_σ_* *α_δ_*, and *α_η_*. A sum of these coefficients is unity. We arbitrarily determine the importance of these coefficients depending on our sensing purposes. By using weight coefficients, we determine an optimum value, *n_opt_*, as follows:

(10) $$n_{\mathrm{opt}}=\alpha_{\sigma }\cdot \mathrm{norm}\sigma +\alpha_{\delta }\cdot \mathrm{norm}\delta +\alpha_{\eta }\cdot \mathrm{norm}\eta$$

Equation (10) tells us that the maximum *n_opt_* gives an optimum dipping duration for given weight coefficients. If we need to maximize *σ* but neglect the other factors, we can set *α_σ_* as unity and others as zero. This condition is labeled as condition *1, and *n_opt_* is listed in Table 3. In this weight condition, AAPSP_100_ gives an optimum. If we design an AA-PSP such that all three outputs are equally important, we set *α_σ_*, *α_δ_*, and *α_η_* as 1/3. The value *n_opt_* was listed in Table 3 as condition *2. In this weight condition, AAPSP_3600_ gives an optimum. By introducing *n_opt_*, we can design an AA-PSP for our sensing purposes related to the dipping duration.

### 4.2. Repeatability

The errors shown in Table 2 were caused by the differences in the dipping deposition. Factors causing the errors may be the luminophore concentration, dipping duration, and dipping temperature. These factors can be minimized by preparing an AA-PSP at the same time. However, to discuss the repeatability of AA-PSP preparation, each AA-PSP was dipped separately. Because we used the same luminophore solution, the first factor can be fairly neglected. Even though carefully controlled, ±1 s difference in the dipping duration would be considered. Based on the presented results, this difference may not greatly influence to the pressure sensitivity, but the error may appear to the temperature dependency and signal level. The third factor was fixed at 25 °C in our experiment. There may be a small variation in a temperature control during the dipping process. This would be a factor in the dipping deposition to influence the AA-PSP characterizations. Another factor besides the dipping deposition to cause the error may be the calibration fitting error. It is related to the determination of calibration constants, which is directly related to the AA-PSP characterizations. This error can be minimized by increasing calibration points. Sakaue and Ishii reported the error estimation of the repeating cycle of pressure and temperature calibrations for a given AA-PSP [3]. AAPSP_3600_ was calibrated repeatedly by increasing and decreasing the pressures and temperatures. The pressure sensitivity showed ±0.3% error, and the temperature dependency showed ±0.6 %/°C error. The preparation procedure gave one order magnitude in error for the pressure sensitivity, while for the temperature dependency, both error sources showed the same order of magnitude.

### 4.3. AA-PSP Characterizations related to Dipping Parameters

Previous studies reported the effects of the dipping parameters besides the dipping duration on the AA-PSP characterizations [2,3]. These were the solvent dependency and luminophore concentration. In total eight solvents (hexane, toluene, dichloromethane, chloroform, acetone, *N*,*N*-dimethylformamide, dimethylsulfoxide, and water) were selected for the solvent dependency studies in order from non-polar to the highest polarity index. The luminophore concentration was selected from a very dilute case of 0.001 mM to 10 mM, where the luminophore reached to its saturation. In the present case, we varied the dipping duration from a very short dipping of 1 s to a very long dipping of 100,000 s (over 1 day). Even though the upper limit of the dipping duration would be infinity, we assumed that over 1 day of dipping duration would be enough to understand the change in the AA-PSP characterizations. Table 4 lists the maximum and minimum values of AA-PSP characterizations reported from references [2,3]. Here, *η* was based on the reference AA-PSP of AAPSP_3600_.

The pressure sensitivity was greatly influenced by the solvent. The difference in the sensitivity was a factor of 10.3. By varying the luminophore concentration, the difference was a factor of 2. As shown in Section 3.1., the difference was a factor of 1.2 by varying the dipping duration. Among the dipping parameters, the pressure sensitivity was most influenced by the solvent.

The temperature dependency was not reported in the reference [2]. Compared to the present results (Section 3.2.) and the reference [3], the difference in the temperature dependency was on the same order, which was a factor of 2. The temperature dependency was influenced by the dipping parameters, but the change was not as large as that of the pressure sensitivity.

The signal level was greatly influenced by varying the solvent. The difference was a factor of 14.5. The second largest effect was the dipping duration. The difference in the signal level was a factor of 8.4. The luminophore concentration influenced the signal level for a factor of 3.6. Overall, the signal level was most influenced by the dipping parameters.

## 5. Conclusions

This paper discussed the parameters in the dipping deposition method for optimizing anodized-aluminum pressure-sensitive paint (AA-PSP). The parameters were the dipping duration, dipping solvent, and luminophore concentration. The first parameter was varied from 1 to 100,000 s relating to the AA-PSP characterizations of the pressure sensitivity, temperature dependency, and signal level. The maximum pressure sensitivity was obtained at the dipping duration of 100 s, the minimum temperature dependency was obtained at the duration of 1 s, and the maximum signal level was obtained at the duration of 1,000 s, respectively. Among the characterizations, the dipping duration most influenced the signal level, which showed the difference of the signal level by a factor of 8.4. By introducing a weight coefficient, an optimum dipping duration can be determined. Among all the dipping parameters, it was found that the pressure sensitivity and signal level were most influenced by the dipping solvent.

## Figures and Tables

**Table 1.** Dipping conditions of AA-PSP. Bathophen ruthenium was chosen as a luminophore and dichloromethane was chosen as a solvent. The luminophore concentration was fixed at 0.1 mM.

**Sample ID**	**Dipping Duration (s)**
AAPSP_1_	1
AAPSP_10_	10
AAPSP_100_	100
AAPSP_1000_	1,000
AAPSP_3600_	3,600
AAPSP_100000_	100,000

**Table 2.** Summary of AA-PSP characterizations. The error was determined as the standard deviation of the three data sets from the same dipping procedures.

**Sample ID**	**Pressure Sensitivity *σ* (%)**	**Temperature Dependency *δ* (%/°C)**	**Signal Level *η* (%)**
AAPSP_1_	52 ± 1	−1.10 ± 0.10	20.0 ± 2.8
AAPSP_10_	56 ± 2	−1.63 ± 0.30	45.2 ± 6.8
AAPSP_100_	65 ± 2	−2.35 ± 0.22	139.4 ± 20.4
AAPSP_1000_	63 ± 2	−2.11 ± 0.11	167.1 ± 17.1
AAPSP_3600_	62 ± 4	−1.38 ± 0.15	100.0 ± 14.8
AAPSP_100000_	59 ± 4	−1.28 ± 0.21	67.2 ± 11.2

**Table 3.** Optimum value, *n_opt_*, determined from weight coefficients, *α_σ_*, *α_δ_*, and *α_η_* for given dipping duration. Condition *1: *α_σ_* = 1 and others are zero. Condition *2: *α_σ_* = *α_δ_* = *α_η_* = 1/3.

**Sample ID**	*n_opt_* *1	*n_opt_* *2
AAPSP_1_	0.00	0.33
AAPSP_10_	0.30	0.34
AAPSP_100_	1.00	0.60
AAPSP_1000_	0.85	0.68
AAPSP_3600_	0.82	0.71
AAPSP_100000_	0.56	0.58

**Table 4.** The maximum and minimum AA-PSP characterizations reported from Refs. [2,3]. The results from the present case were also listed.

	**Polarity Index**		**Luminophore Concentration**		**Dipping Duration**	

	**max.**	**min.**	**max.**	**min.**	**max.**	**min.**
*σ* (%)	62	6	62	31	65	52
*δ* (%/°C)	NA	NA	−0.62	−1.44	−1.10	−2.35
*η* (%)	189	13	100	27.5	167.1	20.1
